# Maternal-fetal interface abnormalities in unexplained recurrent pregnancy loss: mechanisms, models, and a framework for subtype identification

**DOI:** 10.3389/fendo.2026.1900874

**Published:** 2026-07-30

**Authors:** Asahi Tokuoka, Kiriko Iida, Mitsutoshi Yamada, Masanori Ono, Naoko Irie

**Affiliations:** 1 Department of Obstetrics and Gynecology, Keio University School of Medicine, Tokyo, Japan; 2 Department of Molecular Biology, Keio University School of Medicine, Tokyo, Japan

**Keywords:** decidua, endometrium, immune regulation, maternal-fetal interface, trophoblast, unexplained recurrent pregnancy loss

## Abstract

Recurrent pregnancy loss (RPL) is a heterogeneous reproductive condition, and unexplained recurrent pregnancy loss (URPL) denotes repeated pregnancy losses for which no established cause is identified after standard evaluation. Recurrent miscarriage (RM) and recurrent spontaneous abortion (RSA) are overlapping terms used in the literature, whereas recurrent implantation failure (RIF) is a related but distinct infertility context. In this Mini Review, URPL is treated as an exclusion-based clinical category rather than a single disease entity. This review examines the hypothesis that a subset of URPL reflects abnormalities in specific cellular and molecular components of the maternal-fetal interface. We focus on maternal compartments, including endometrial epithelial lineage states, decidual stromal dysfunction, and stromal-immune interactions, as well as fetal/trophoblast compartments such as trophoblast lineage defects and broader maternal-fetal interactions, incorporating insights from recent advances in molecular and cellular technologies and human model systems. Particular attention is given to single-cell, spatial, organoid, assembloid, implantation model, trophoblast stem cell, and placental explant studies published from 2024 to 2026. Current data do not establish clinical subtypes for URPL or support treatment selection in routine practice. However, recent molecular and cellular technologies now make it possible to define interface phenotypes with higher resolution and to test whether patient-derived abnormalities are reproducible and perturbable in experimental systems. An interface-centered framework may therefore help move URPL research from broad exclusion-based labels toward experimentally testable subtype identification based on epithelial, decidual, trophoblast, immune, and embryo-endometrial phenotypes.

## 1 Introduction

Recurrent pregnancy loss (RPL) is a clinically important reproductive problem, but its definition and biological interpretation remain unsettled. The European Society of Human Reproduction and Embryology (ESHRE) guideline states that a diagnosis of RPL can be considered after the loss of two or more pregnancies, while also emphasizing that the evidence base remains limited and that definitions and management strategies are heterogeneous (1, 2). In this review, RPL is used as the broad umbrella term when discussing guideline frameworks or studies that did not restrict enrollment to unexplained cases. Unexplained recurrent pregnancy loss (URPL) is used as a working term for repeated pregnancy losses in which standard evaluation does not identify an established cause. Standard evaluation generally considers parental or embryonic chromosomal abnormalities, uterine anatomical factors, antiphospholipid syndrome, selected endocrine disorders, and other clinically actionable conditions.

Several related terms require clarification for non-expert readers. Recurrent miscarriage (RM) and recurrent spontaneous abortion (RSA) are overlapping terms that appear in different clinical and research traditions. They are broader than URPL when studies include both explained and unexplained cases. Recurrent implantation failure (RIF) is not synonymous with RPL or URPL because it refers to failure to achieve implantation after embryo transfer. However, RIF studies may provide indirect mechanistic evidence for embryo-endometrial interactions. In this review, the original terms used by each cited study are retained, but evidence from RM, RSA, RIF, normal pregnancy atlases, and experimental models is treated as indirect for URPL unless the study specifically enrolled unexplained RPL or URPL cohorts.

URPL is therefore an exclusion-based clinical category, not a disease defined by one mechanism. Reviews have framed RPL as a heterogeneous condition in which anatomical, endocrine, genetic, immune, endometrial, and placental factors may contribute with variable weight across patients (3, 4). Previous immunological reviews, including the framework proposed by Vomstein et al., had already highlighted candidate immune-related factors in RPL, such as NK cells, regulatory T cells, dendritic cells, plasma cells, chronic endometritis, and HLA-related alloimmune mechanisms (5). A more useful question is whether reproducible biological subtypes can be identified within the unexplained group. The maternal-fetal interface provides a practical framework for asking this question.

**Figure 1:**
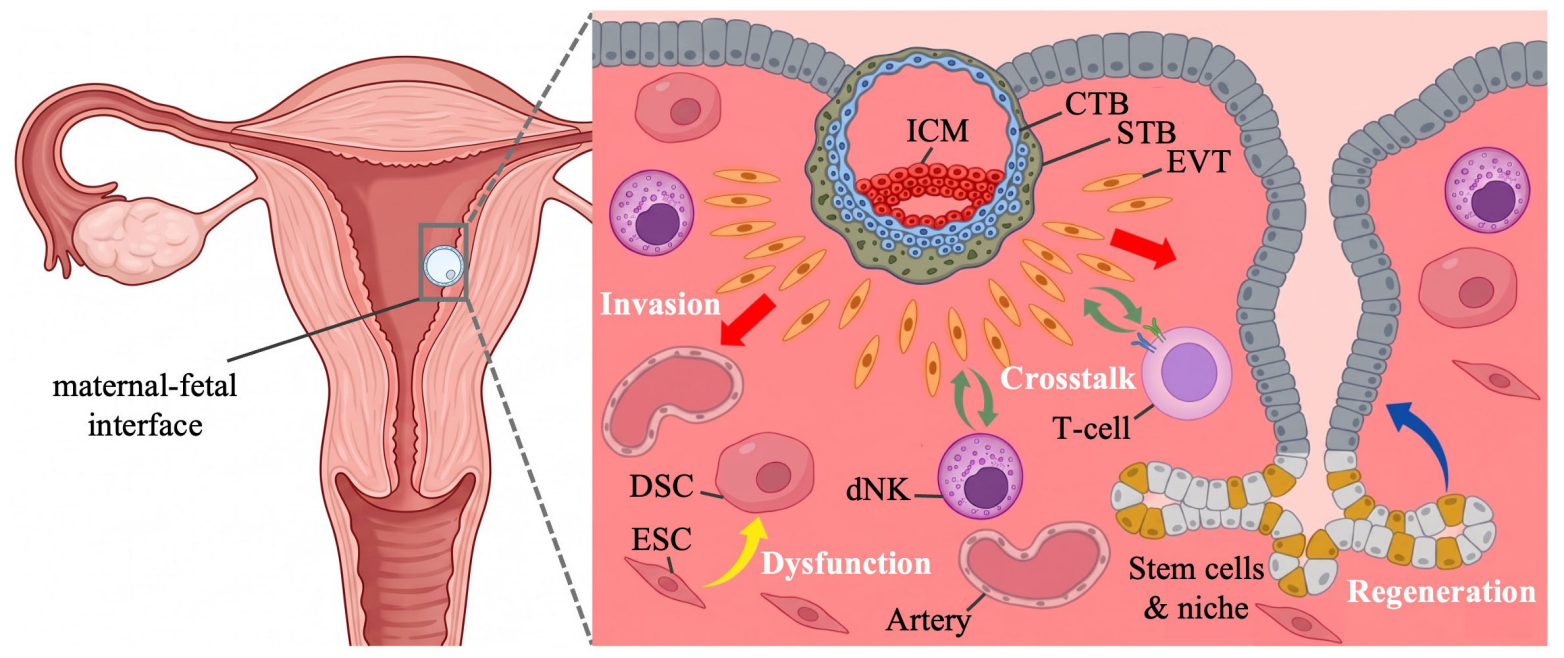
Candidate maternal-fetal interface phenotypes across stages of implantation and early placentation. The schematic is structured to show candidate domains across maternal and fetal compartments. Maternal domains include preconception endometrial epithelial lineage and cyclical regeneration, decidual stromal dysfunction, and stromal-immune crosstalk. Fetal/trophoblast domains include trophoblast lineage dysfunction and extravillous trophoblast invasion. The image is intended as a research framework and does not indicate established URPL subtypes. ICM, inner cell mass; CTB, cytotrophoblast; STB, syncytiotrophoblast; EVT, extravillous trophoblast; dNK, decidual natural killer cell; DSC, decidual stromal cell; ESC, endometrial stromal cell.

The maternal-fetal interface includes both maternal and fetal compartments. The maternal side includes the endometrial epithelium, decidual stromal cells, immune cells, vascular cells, and local endocrine signals. The fetal side includes trophoblast lineages derived from the embryo, including cytotrophoblasts, syncytiotrophoblasts, and extravillous trophoblasts. Implantation and early placentation require coordinated interactions across these compartments. This framework does not imply that all URPL is caused by interface failure. Rather, it defines testable domains, including epithelial regeneration and receptivity, decidual reaction, trophoblast differentiation and invasion, stromal-immune communication, and embryo-endometrial signaling (6). **Figure 1** maps these candidate phenotypes onto a staging axis from the preconception endometrium to early placentation. **Table 1** summarizes representative recent literature.

**Table 1:** Representative recent literature and human model systems relevant to maternal-fetal interface phenotypes.

Domain	Key studies	Experimental or clinical material	Main finding	Clinical or experimental setting	Relevance and limitations for URPL research
Endometrial epithelial lineage and receptivity	Mareckova M. Nat Genet. 2024 (7)	Human Endometrial Cell Atlas; 63 women; 313,527 cells; spatial validation	Defines epithelial and stromal cell states and basalis signaling involving SOX9+ epithelial populations.	Normal endometrial reference atlas	Not URPL specific. Useful as a normal reference for epithelial and stromal cell states.
	Al-Lamee H. FASEB J. 2025 (8)	SSEA-1+ endometrial epithelial cells from human biopsies	SSEA-1+ cells showed programs related to regeneration, remodeling, and homeostasis.	Endometrial progenitor biology	Not URPL specific. Provides epithelial progenitor programs that can be tested in URPL studies.
Decidual stromal dysfunction	Muter J. Sci Adv. 2025 (10)	Clinical and molecular analysis of miscarriage	RM risk aligned more closely with stalled or weakened decidual reaction than with poor uNK expansion.	RM with endometrial phenotyping	Relevant to RM and decidual stromal phenotyping. Relevant to URPL if established causes were excluded.
	Chen Y. Cell Metab. 2026 (15)	Human endometrial stromal cells, decidual tissue, and mouse models	Endometrial stromal cell-derived TMAO promoted decidualization via 14-3-3eta/PDK1/FOXO1.	RSA focused human and mouse mechanistic study	Relevant to RSA and mechanistic. Relevant to URPL if established causes were excluded.
Trophoblast lineage dysfunction	Zhang L. Cell Death Dis. 2026 (17)	RPL and normal chorionic villi-decidua pairs; scRNA-seq, glycoproteomics, organ-on-chip	Siglec-7+ dNK cells to stimulate IL-8, activate STAT3 in EVTs, and support invasion; restoring sialylation.	RPL associated trophoblast-immune study	Relevant to RPL and EVT invasion to immune-related ligand-receptor signaling. Relevant to URPL if established causes were excluded.
	Wu Z. Proc Natl Acad Sci. 2025 (18)	RSA villi, abortion-prone mice, hTSCs, immortalized EVT-like model	MYBL2 downregulation impaired hTSC stemness and EVT lineage development through AJUBA/Hippo-related mechanisms.	RSA focused trophoblast lineage mechanistic study	Relevant to RSA and Useful for testing trophoblast-lineage defects.
Stromal-immune crosstalk at the maternal-fetal interface	Wang C. Nature. 2026 (24)	221,380 snRNA-seq, 210,191 snATAC-seq, spatial whole-transcriptomic maps across gestational week 5-39	Mapped cell states, trophoblast and DSC differentiation, arterial remodeling, and miscarriage-vulnerable cell states.	Normal maternal-fetal interface atlas	Not URPL specific. Provides a spatial and temporal reference for pathological samples.
	Wang D. Front Immunol. 2026 (26)	Unexplained spontaneous abortion decidua, DSC-pNK/dNK co-culture, HAS2 knockdown DSCs, CD44 blockade	DSC-derived HA/HAS2-CD44-Wnt-FOSL2 signaling promoted dNK1-like differentiation and immune tolerance.	Unexplained pregnancy loss stromal-immune study	Supports the concept that stromal products instruct immune states at the interface; requires URPL-specific validation.
Human model systems	Shibata S. Sci Adv. 2024 (27)	Hormone-responsive apical-out endometrial organoids with exposed apical epithelium, stromal cells, and endothelial network	Recapitulated apposition, adhesion, invasion, and syncytial trophoblast interaction with endometrial stromal cells.	Human implantation model	Not URPL specific. Useful as a basis for epithelial-facing co-culture and early trophoblast interaction studies.
	Mole M. Cell. 2026 (28)	Cell-engineered Receptive Endometrial Scaffold Technology reconstructing luminal, glandular, and stromal compartments with human embryos or blastoids	Modeled implantation and early post-implantation hallmarks within a receptive endometrial scaffold.	Integrated human implantation model	Not URPL specific. URPL specific use may be possible with well-defined patient-derived endometrial or decidual tissue.
	Li Q. Cell. 2026 (29)	3D in-chip model using human blastoids or blastocysts with control- or RIF-derived bioengineered endometrioids	Recapitulated apposition, attachment, invasion, and early post-implantation development; RIF-derived endometrioids showed impaired implantation.	RIF associated implantation model	RIF related, not URPL specific. URPL use requires strictly defined URPL-derived endometrioids and appropriate comparators.
	Song J. Cell Stem Cell. 2026 (30)	3D co-culture platform using human embryos and endometrial organoids	Enabled reciprocal embryo-endometrial communication and modeled peri- and post-implantation development.	Human post-implantation co-culture model	Not URPL specific. Useful as a basis for 3D embryo-endometrial communication studies.
	Zhang Y. Elife. 2026 (31)	Human window-of-implantation endometrial assembloids with receptive epithelial and stromal features	Reproduced receptive endometrial features, including pinopode-like structures, cilia, hormone responsiveness, and implantation potential.	Endometrial receptivity model	Not URPL specific. Useful for scalable receptivity phenotyping.
	Yoneda T. Reprod Med Biol. 2026 (32)	ECM-free human endometrial organoids with apical-out polarity and an inner stromal compartment	Established hormone-responsive organoids with epithelial polarity, stromal organization, and receptivity-related marker expression.	Endometrial organoid platform	Not URPL specific. Useful for scalable epithelial-stromal hormone-response testing.
	Ruiz-Morales E. STAR Protoc. 2025 (33)	Early-pregnancy human placental explants at the air-liquid interface for pathogen exposure, perturbation assays, and CD45-positive cell enrichment	Preserved trophoblast and Hofbauer cell features and enabled short term infection and immune cell enrichment assays.	Acute pathogen response placental explant protocol	Not URPL specific. Useful for infection- or inflammation-associated trophoblast-immune perturbation studies.

URPL, unexplained recurrent pregnancy loss; RM, recurrent miscarriage; uNK, uterine natural killer cell; RSA, recurrent spontaneous abortion; RPL, recurrent pregnancy loss; scRNA-seq, single-cell RNA sequencing; dNK, decidual natural killer cell; EVT, extravillous trophoblast; hTSC, human trophoblast stem cell; snRNA-seq, single-nucleus RNA sequencing; snATAC-seq, single-nucleus assay for transposase-accessible chromatin with high-throughput sequencing; DSC, decidual stromal cell; RIF, recurrent implantation failure; ECM, extracellular matrix.

Recent technological advances have substantially expanded the ability to investigate the human maternal-fetal interface at molecular and cellular resolution. Single-cell and spatial transcriptomic approaches, together with emerging multiomic technologies, now permit characterization of epithelial, stromal, immune, and trophoblast cell states within their native tissue context. In parallel, advances in human pluripotent stem cell technologies, organoid and assembloid systems, implantation models, trophoblast stem cell systems, and placental explant culture have enabled experimentally tractable platforms for studying early human development and embryo-endometrial interactions. These approaches, together with increasing access to ethically obtained donated human embryonic, fetal, placental, and decidual tissues are beginning to provide mechanistic insight into the cellular and molecular basis of implantation failure, defective placentation, and pregnancy loss.

For transparency, the literature was selected through a narrative search of PubMed/MEDLINE performed on April 28, 2026. The coverage period was January 1, 2014, to April 28, 2026. Search terms included combinations of recurrent pregnancy loss, unexplained recurrent pregnancy loss, recurrent miscarriage, recurrent spontaneous abortion, maternal-fetal interface, decidua, trophoblast, endometrium, immune tolerance, single-cell, spatial transcriptomics, organoid, assembloid, and implantation model. Human studies were prioritized. Non-URPL model systems were included when they provided essential mechanistic context or experimentally testable platforms. The selection was narrative rather than systematic, and the manuscript therefore emphasizes conceptual synthesis rather than exhaustive evidence grading. Because this Mini Review focuses on recent molecular, cellular, and human model-system advances, studies published mainly from 2024 to 2026 were prioritized. Selected foundational studies from 2014 onward were included when they established concepts required to interpret recent work, such as decidual senescence, trophoblast invasion, immune tolerance, epithelial lineage states, or human endometrial model systems.

## 2 Preconception endometrial epithelial lineage and cyclical regeneration

Endometrial epithelial cells form the first maternal compartment encountered by the implanting embryo. The luminal epithelium mediates apposition and attachment, whereas the glandular epithelium produces secretory signals that support early implantation. These differentiated epithelial states arise within a tissue that undergoes repeated regeneration during reproductive life. Therefore, epithelial receptivity should not be viewed only as a terminal secretory-phase state. It may also reflect the quality of upstream epithelial lineage maintenance and cyclical regeneration.

Recent endometrial atlas studies have revealed extensive spatiotemporal heterogeneity of epithelial and stromal cell states across the menstrual cycle and peri-implantation period. Mareckova et al. generated the Human Endometrial Cell Atlas, a high-resolution reference based on single-cell transcriptomic datasets from 63 women and 313, 527 cells (7). The atlas identified previously unreported cell states and mapped them *in situ* using spatial transcriptomics. In the basalis, the study defined signaling between fibroblasts and an epithelial population expressing progenitor marker SOX9. Al-Lamee et al. profiled SSEA-1-positive endometrial epithelial progenitor cells and highlighted transcriptional programs related to regeneration, remodeling, and homeostasis (8). These studies support the concept that surface receptivity may depend partly on stem/progenitor-like states maintained within a stromal-epithelial niche.

The available atlas and progenitor studies were not designed to establish epithelial progenitor dysfunction as a cause of URPL. Rather, they define measurable epithelial states that can be tested in future URPL studies. Future studies should therefore compare epithelial lineage states across carefully defined URPL, explained RPL, infertility, and fertile control cohorts before assigning causal weight to this domain.

## 3 Decidual stromal dysfunction as a candidate interface phenotype

After epithelial regeneration and acquisition of receptivity, decidual stromal transformation provides the next major maternal compartment required for implantation. During decidualization, endometrial stromal cells undergo progesterone- and cyclic AMP-dependent differentiation and acquire secretory, structural, and immunoregulatory functions that support implantation and early placentation (9). In URPL research, this domain is important because a decidual defect may exist before conception or during the peri-implantation window. Such a defect may therefore represent a recurrent endometrial phenotype rather than only a consequence of pregnancy loss.

Recent work has strengthened the link between decidual reaction and recurrence risk. Muter et al. reported that miscarriage recurrence risk aligned more closely with a weakened or stalled decidual reaction than with poor uterine natural killer cell expansion (10). This observation shifts attention from immune-cell counts alone to the stromal program that organizes the peri-implantation environment. Earlier model-based work is consistent with this interpretation. Lucas et al. described an association between RPL and a pro-senescent decidual response during the peri-implantation window (11), and Rawlings et al. showed that endometrial assembloids can model decidual senescence and its effects on embryo implantation behavior (12). Together, these studies suggest that part of RPL risk may arise from persistent stromal states that alter the implantation niche.

Newer studies divide decidual dysfunction into more specific spatial, cellular, and metabolic phenotypes. Sha et al. used spatial transcriptomics of human decidua to identify molecular signatures in RPL, providing a spatial reference for decidual domains and implantation-zone abnormalities (13). Qin et al. identified CD24-positive decidual stromal cells as a heterogeneous stromal population associated with impaired regulatory T-cell induction in RM (14). Chen et al. reported that endometrial stromal cell-derived trimethylamine N-oxide supported decidualization through 14-3–3 eta, PDK1, and FOXO1-related mechanisms in RSA models (15). These studies suggest that stromal dysfunction in RPL is unlikely to represent a single abnormality.

Together, these studies support a practical research direction. Patients with URPL may be stratified by measurable preconception or peri-implantation stromal phenotypes, including stalled decidual reaction, senescence-associated states, altered stromal subpopulations, or metabolic decidualization defects. This interpretation remains provisional because embryonic chromosomal status, gestational age, sampling timing, assisted reproductive technology exposure, maternal background, medication exposure, and inflammation may influence tissue profiles. Future URPL studies should therefore pair tissue phenotyping with detailed clinical annotation and, when possible, embryonic genetic information.

## 4 Fetal compartment: trophoblast lineage dysfunction and extravillous trophoblast invasion

Embryo-derived trophoblast cells arise from the outer layer of the blastocyst and give rise to placental lineages. They represent a key fetal compartment of the maternal-fetal interface. Cytotrophoblasts (CTB) are proliferative trophoblast progenitors, syncytiotrophoblasts (STB) support endocrine and exchange functions, and extravillous trophoblasts (EVT) invade the decidua and participate in uterine remodeling. Defects in these lineage programs may compromise implantation stability and early placentation even when a blastocyst has formed. Earlier work identified candidate pathways involved in trophoblast migration and invasion (16), and recent studies provide more direct trophoblast-lineage examples.

Several recent reports connect trophoblast lineage defects to RPL phenotypes. Zhang et al. reported that impaired sialylation-Siglec-7 interaction reduces extravillous trophoblast invasion and may contribute to RPL (17). Restoration of EVT sialylation re-engaged Siglec-7 signaling, rescued IL-8-STAT3 activity, and improved invasive capacity. Wu et al. reported that dysregulation of MYBL2 impairs EVT lineage development and function in RSA (18). Yang et al. used single-cell analysis of the maternal-fetal interface and reported loss of dNK1/2 and EVT1 cells in RM (19). Vasconcelos et al. analyzed products of conception using transcriptomic and deconvolution approaches and identified reduced STB representation, altered CTB and EVT signals, immune pathway enrichment, and histological chronic histiocytic intervillositis in idiopathic RPL (20). These studies place trophoblast dysfunction within a broader interface network rather than as an isolated fetal-side defect.

Patient-specific trophoblast systems may help separate intrinsic trophoblast defects from secondary changes after pregnancy failure. Varberg et al. showed that trophoblast stem cell lines can be derived from chorionic villus tissue and differentiated toward STB and EVT lineages (21). Kim et al. highlighted the potential of patient-specific trophoblast stem cells for studying placental disease mechanisms and individualized risk (22). These models do not eliminate the limitations of post-loss tissue, but they allow investigators to test whether abnormal trophoblast differentiation or invasion persists after derivation and controlled culture. Persistence of a phenotype would support, but not prove, an intrinsic trophoblast-lineage abnormality. Loss of the phenotype in culture would suggest that the tissue profile may mainly reflect the failed pregnancy environment.

## 5 Maternal-fetal stromal-immune crosstalk

Immune abnormalities have long been discussed in RPL, but they should not be interpreted separately from the anatomical organization of the maternal-fetal interface. Kareus et al. described the maternal-fetal interface as a specialized immunological barrier composed of the decidua, intervillous space, and fetal membranes, each forming a distinct site of maternal-fetal interaction (23). This spatial view is useful for URPL research because immune findings may arise from different compartments and stages rather than from a single systemic immune imbalance.

Early gestation depends on coordinated immune adaptation. Kareus et al. summarized evidence that macrophages, regulatory T cells, and uterine natural killer cells support implantation, tolerance, trophoblast invasion, and vascular remodeling (23). Recent single-cell and spatial studies also support this network view. Wang et al. reported a spatiotemporal dissection of the human maternal-fetal interface across gestation, integrating transcriptomic, chromatin, spatial, and protein-level information that can help map pathological samples onto normal gestational trajectories (24). Qiu et al. described a Rev-erbα - GFPT1 - CD58 - CD2 axis that regulates communication between decidual stromal cells and decidual CD4-positive T cells (25). Wang et al. described a hyaluronic acid-HAS2-CD44-Wnt-FOSL2 axis through which decidual stromal cells promote dNK1-like differentiation, reduce cytotoxicity, and support immune tolerance (26). These studies suggest that immune findings should be interpreted together with epithelial, stromal, trophoblast, and placental compartment states.

The trophoblast side supports the same logic. In the sialylation-Siglec-7 pathway, the immune-related signal is relevant because it modulates EVT invasion rather than representing immune imbalance alone (17). Macrophage, uterine natural killer cell, and T-cell phenotypes may therefore reflect a primary immune defect, a response to abnormal decidualization, or a consequence of altered trophoblast signaling.

## 6 Human model systems for maternal-fetal interface interactions

Human implantation is difficult to study directly because the earliest stages occur within the endometrium and are rarely accessible for dynamic observation. Human model systems are therefore important because they allow investigators to move from descriptive association to perturbation-based testing. For URPL research, however, these systems should not be interpreted as interchangeable models of pregnancy loss. Their value depends on which compartment they capture, whether patient-specific phenotypes can be reproduced, and whether the observed abnormality can be linked back to clinically defined URPL cohorts. **Table 1** summarizes the original study context and the relevance and limitations of these platforms for research focused on URPL.

Recent integrated models are important because they move the field from descriptive association to perturbation-based testing. Shibata et al. modeled the embryo-endometrial interface using apical-out endometrial organoids (27). This system provides an embryo-facing epithelial surface and is useful for testing epithelial polarity, receptivity-related responses, embryo attachment, and early trophoblast interaction. Mole et al. developed Cell-engineered Receptive Endometrial Scaffold Technology (CREST), which reconstructs luminal, glandular, and stromal compartments of receptive human endometrium (28). Human embryos and blastoids implanted into this scaffold and showed post-implantation features, including trophoblast proliferation, STB differentiation, EVT migration, and early placental structures.

Patient-derived systems add another level of relevance because they can test whether a phenotype is reproducible in cells or tissues from a specific clinical group. Li et al. developed a three-dimensional in-chip model of implantation using human blastoids or blastocysts and bioengineered endometrioids (29). In endometrioids derived from patients with RIF, blastoid implantation capability was reduced compared with controls, and compound screening identified candidates that improved implantation efficiency in selected patient-derived models. This platform is important for research focused on URPL because it illustrates patient-derived and perturbable testing of the endometrial side. However, its current evidence derives from RIF rather than URPL. Application to URPL would require endometrioids derived from strictly defined URPL cohorts.

Other integrated endometrial models provide complementary strengths. Song et al. established a three-dimensional post-implantation co-culture of human embryo and endometrium (30). Zhang et al. developed a human receptive endometrial assembloid to study the implantation window, including hormone responsiveness, receptive epithelial states, cilia, pinopode-like structures, and embryo implantation potential (31). Yoneda et al. reported an extracellular matrix-free human endometrial organoid with apical-out epithelial polarity, an inner stromal compartment, and hormone-responsive expression of receptivity-related markers (32). These systems are useful for testing epithelial maturation, stromal organization, and embryo-endometrial signaling. Their main limitation for URPL research is that they do not yet capture the full trophoblast, immune, vascular, and clinical heterogeneity of recurrent pregnancy loss.

Placental explant systems provide a complementary approach for studying the placental side of the interface. Ruiz-Morales et al. presented an air-liquid interface protocol for early-pregnancy human placental explants that maintains key trophoblast and Hofbauer cell traits and preserves multilineage viability for short-term perturbation studies (33). The protocol supports infection assays, tissue dissociation, single-cell suspension preparation, and CD45-positive immune-cell enrichment. Although this platform is not an endometrial model and was designed for acute pathogen responses rather than URPL, it is relevant to interface phenotyping because it illustrates how infection-induced inflammatory perturbation can be introduced into human tissue-based models. This concept may inform future co-culture studies that use inflamed endometrial or decidual components to test whether inflammatory states alter embryo-endometrial or trophoblast-immune interactions.

In this review, responsiveness refers to model-based responsiveness rather than established clinical treatment response. If patient-derived phenotypes such as impaired epithelial maturation, impaired decidualization, reduced trophoblast invasion, or defective embryo-endometrial signaling can be measured and partly rescued in a model system, that response may help define a biologically meaningful interface phenotype. These models provide a framework for moving beyond non-stratified empirical management. Human model systems therefore have value because they make interface phenotypes measurable, perturbable, and comparable across patients.

## 7 Discussion

The central synthesis is that maternal compartments, fetal/trophoblast compartments, and their interactions should be examined as connected domains rather than isolated mechanisms. On the maternal side, relevant domains include epithelial lineage maintenance, cyclical regeneration, decidual stromal transformation, and stromal-immune communication. On the fetal side, relevant domains include trophoblast lineage development, syncytiotrophoblast differentiation, and extravillous trophoblast invasion. This framework does not replace guideline based evaluation for established causes of RPL. It addresses the remaining problem that many patients have repeated losses without a recognized diagnosis after standard evaluation.

URPL should be interpreted as an exclusion-based residual category, not as a single disease entity. This distinction is important when data from RPL, RM, RSA, RIF, and normal pregnancy atlases are interpreted together. The studies reviewed here indicate that maternal-fetal interface abnormalities may arise from several connected compartments, including the endometrial epithelium, decidual stroma, trophoblast lineages, immune cells, and embryo-endometrial interaction. However, current evidence does not establish clinically actionable URPL subtypes. Instead, it supports a research framework in which URPL can be decomposed into experimentally testable interface phenotypes.

Terminological heterogeneity remains a major limitation in this field. URPL, unexplained RPL, idiopathic RPL, RPL, RM, RSA, and RIF are often used across partially overlapping but clinically distinct populations. These differences affect study composition, diagnostic exclusion, gestational age, embryonic chromosomal status, and exposure to assisted reproductive technology. Therefore, findings from RPL, RM, RSA, or RIF studies should be interpreted as a heterogeneous evidence base rather than as directly equivalent URPL evidence. Future studies should report the diagnostic workup used to define unexplained cases, specify whether embryonic chromosomal testing was performed, and stratify analyses by gestational age, ART exposure, medication exposure, and inflammatory or infectious findings when possible.

The reviewed studies form a layered evidence base. Direct clinical evidence for URPL specific interface phenotypes remains limited. Evidence from RPL, RM, RSA, and idiopathic pregnancy loss studies provide disease-adjacent signals, but these signals are influenced by variation in diagnostic workup and definitions. RIF studies are mechanistically relevant to embryo-endometrial interactions, but they address a distinct clinical context and should not be directly equated with URPL. Normal single-cell and spatial atlases provide reference trajectories, but they do not define disease mechanisms by themselves. Experimental organoid, assembloid, trophoblast stem cell, implantation, and placental explant models provide platforms for perturbation and rescue, but most are not yet validated as URPL models.

Several limitations are important for interpretation. First, URPL is defined by exclusion, and the completeness of evaluation varies across studies. Many mechanistic studies use RM, RSA, RIF, infection models, placental inflammation, or infertility studies rather than strictly defined URPL. Second, miscarriage-derived tissue may contain both initiating abnormalities and downstream consequences of pregnancy failure. Third, key confounders include embryonic chromosomal status, gestational age, sampling timing, assisted reproductive technology use, maternal age and comorbidities, medication exposure, inflammation, and tissue processing. These limitations mean that the proposed framework should be viewed as a research structure rather than a clinical classification.

Despite these limitations, an interface-based framework may help transform URPL from a residual label into a set of experimentally testable phenotypes. Future studies should combine careful clinical phenotyping with patient-derived epithelial, stromal, trophoblast, implantation, and placental explant models. These studies should test whether specific interface abnormalities persist across cycles, predict recurrence, or show reproducible model-based responsiveness. This design would help separate recurrent patient-specific phenotypes from transient tissue states that follow a failed pregnancy.

In conclusion, recent maternal-fetal interface research provides a useful route for defining experimentally testable interface phenotypes in URPL. The most defensible current position is not that URPL is caused by one interface defect. Rather, selected patients may have measurable abnormalities in epithelial, decidual, trophoblast, immune, or embryo-endometrial domains. A compartment-based framework can clarify these domains and help connect clinical URPL cohorts with emerging molecular technologies and human model systems.
